# Dr. Martin’s Pellagra Forum

**Published:** 1916-07

**Authors:** 


### Dr. Martin's Pellagra Forum.

It gives us a great deal of pleasure to introduce to our readers Dr. Edward H. Martin, of Hot Springs, Arkansas, as the editor of the Pellagra Forum.

Dr. Martin graduated in the Medical College of Chicago of Ohio in '87. He located in Mississippi, and for twenty-years practiced in the Mississippi Delta, in Coahoma county. The subject of malaria was uppermost in the minds of the physicians of that day, and especially were they interested in the so-called malaria hematuria or black water fever.

Dr. Martin was a leader in the fight against the use of quinine in hematuria, and was instrumental in establishing the fact that quinine is not needed after the explosion of hemolysis takes place. The fight against the use of quinine was a hard one, and it took several years to prove the theory, but so fully did the doctor vindicate his position that no physician in Mississippi today will prescribe quinine in such a condition.

Dr. Martin is an ex-president of the Mississippi State Medical Association, having been president of that State Association before moving to Arkansas.

For many years the doctor has been interested in pellagra, and has been prominently identified with the subject. His first paper on the subject was read before the Tri-State Medical Association of Mississippi, Arkansas and Tennessee at Memphis, November, 1910. This paper introduced the use of soamin in the treatment of pellagra. The next paper was on "The Specific Treatment of Pellagra," and advocated the use of salvarsin. This was read in Oklahoma City in October, 1911. The following year at the Pellagra Conference the same subject was elaborated in a paper having the same title. This was in November, 1912. In May, 1914, the treatment of pellagra was again brought out in a paper read before the Louisiana State Medical Association in New Orleans.

We feel that we are extremely fortunate in having the doctor in charge of the pellagra forum. It is in line with the JOURNAL'S policy of giving its readers the very best information obtainable.

[Please remember that if you have any theories regarding pellagra, or if you wish to ask any questions regarding other theories, you are most welcome to do so.

Address all communications relating to this subject to Dr. E. H. Martin, Hot Springs, Arkansas.]

